# Frequency and characteristics of functional neurological disorder in the emergency department

**DOI:** 10.1007/s00415-025-13386-5

**Published:** 2025-09-22

**Authors:** Kay Hellwig, Benedikt Frank, Rosa Michaelis, Christoph Kleinschnitz, Stoyan Popkirov

**Affiliations:** 1 Department of Neurology and Center for Translational Neuro- and Behavioral Sciences (C-TNBS), University Hospital Essen, University Duisburg-Essen, Essen, Germany; 2 Faculty of Health, University Witten/Herdecke, Witten, Germany; 3 Department of Neurology, University Hospital Knappschaft Kliniken Bochum, Ruhr University Bochum, Bochum, Germany

**Keywords:** Functional neurological disorder, FND, Emergency department, Functional/dissociative seizures, Diagnostic coding

## Abstract

**Background:**

Functional neurological disorders (FND) are common yet frequently under-recognized in emergency settings. Reliance on diagnostic coding alone (e.g. ICD-10 diagnoses F44 and F45) likely underestimates their true prevalence and healthcare impact. This study aimed to assess the frequency and characteristics of FND among adult neurological emergency department (ED) presentations using a hybrid case-finding strategy.

**Methods:**

We conducted a retrospective analysis of all neurological ED presentations (*n* = 5504) to a tertiary university hospital in 2023. A full-text keyword search of 184,279 ED documentation entries was followed by manual review to check the FND-related keywords’ relevance to acute ED presentations. Additional cases were identified from neurological inpatient discharge records. Clinical and demographic variables, presenting symptoms, diagnostic classification, and management pathways were analyzed.

**Results:**

We identified 199 cases of functional symptoms as chief presenting complaints, representing 3.6% of all neurological ED presentations. The patients’ median age was 33 years, and 71% were female. Functional seizures were the most common presentation (54%), followed by motor or sensory deficits (21%). Most patients (75%) were discharged directly from the ED, while 19% were admitted. Discharge against medical advice occurred in 4.5% of FND cases, compared to 1.5% overall. Only 39% of ED-discharged FND cases received F44 or F45 diagnostic codes.

**Conclusions:**

FND is a common cause of neurological emergency presentations. Epidemiological analyses that rely on diagnostic codes risk substantially underestimating its true prevalence. Our findings underscore the need for improved recognition, diagnostic clarity, and structured care pathways for FND in emergency settings.

## Introduction

Functional neurological disorders (FND) are commonly encountered in emergency departments (EDs), yet their frequency and clinical features remain underexplored [1–3]. Large epidemiological studies from the U.S. found that ICD-10 diagnoses F44.4–7 accounted for only 0.5% across millions of neurological ED visits among adults [4, 5]. However, this figure likely represents only a fraction of actual cases of FND, since studies tracking diagnostic outcomes report that 9–13% of ED patients seen by or admitted to neurology are ultimately determined to have had functional symptoms [6, 7]. In studies of specific neurological symptoms among emergency presentations, FND has also been found at high rates: 7–27% of seizures [8–10], 8–13% of dizziness presentations [11, 12], 3–8% of suspected strokes [10, 13, 14], and 20% of acute-onset movement disorders [15].

The discrepancy in incidence between epidemiological analyses and studies tracking individual cases is particularly well illustrated for functional/dissociative seizures. A prospective study in England found that 7.4% of all emergency seizure presentations were eventually diagnosed as functional/dissociative seizures, yet national hospital coding data indicated rates 20 times lower than that [16, 17]. This underrepresentation is likely multifactorial. For one, functional/dissociative seizures are misdiagnosed at high rates at all levels of emergency management [18, 19]. Furthermore, even with correct recognition, there are considerable inconsistencies in diagnostic coding with one study reporting that among 130 patients with prolonged functional/dissociative seizures, there were 48 different diagnostic labels used [20]. Conversely, coding for functional/dissociative seizures can sometimes be applied incorrectly [21]. Both misdiagnosis and inconsistent coding have been noted as significant problems for other forms of FND, too [22, 23]. Thus, the epidemiological and clinical reality of FND in emergency departments has remained largely obscured.

Political and economic decision-making in health care is generally based on analyses of large datasets from tabularized hospital records and insurance claims, rather than small-scale prospective clinical studies. In the case of a neglected and underserved clinical population such as patients with FND, there is no easy way to overcome diagnostic imprecision and coding inconsistency in large datasets without severely limiting the size of the patient sample.

Here, we use a multi-level search, screening and review strategy to identify cases of FND among all emergency department documentation across more than 5000 neurological emergency presentations over an entire year and subsequently characterize relevant cases in demographic and clinical terms. The primary goal was to elucidate the rate of all likely FND coming through the ED, even if they were not coded as such. In addition, we aimed to identify potential for improving diagnostic processes and management.

## Methods

### Case identification

A full-text search of the entire emergency department documentation system was performed for the year 2023 comprising data of 21,926 individual cases seen at the adult non-surgical (medical and neurological) emergency department of the University Hospital Essen. The database included 184,279 individual text entries. As patients under the age of 18 are seen at the hospital’s department of pediatrics with its own emergency and neuropediatric services, this study involves only adult individuals.

Entries containing the letter strings *psychog*, *funktionel*, *disso*, *nicht-epil*, *ychosomat*, *somat* or *Konversions*, aiming to catch all possible German words related to FND including common misspellings and typos, were automatically identified. Other letter strings (including obscure or obsolete medical and colloquial terminology as well as plausible misspellings) were also tried but did not yield any additional hits. Next, identified entries were manually reviewed to check whether the identified search terms were used to describe neurological symptoms or disorders and whether they were related to the current presentation and not, for example, to past medical history.

To complement the dataset with patients whose functional symptoms were not recognized as such until after emergency inpatient admission to the neurological ward, all neurological inpatients with a discharge diagnostic code of F44.x and F45.x (except F45.40 and F45.41, i.e., chronic pain conditions) were identified within the electronic hospital information system (CGM Medico, CGM Clinical Europe Ltd., Germany) and screened for the following inclusion criteria: emergency admission; FND diagnosis related to presenting complaint; not already identified through keyword search of emergency department documentation.

The total number of neurological presentations was extracted from a separate record of all emergency department cases sorted by discipline with available information regarding initial triage and mode of discharge.

### Demographic and clinical characterization

Patients were characterized by age, gender, mode of presentation (ambulance, walk-in, transfer, etc.) and management after emergency department (admission, discharge, transfer, etc.). Triage keywords used for initial assessment were noted. ICD-10 codes were extracted. Presenting symptoms were classified into one of eight categories (sensory or motor deficits, paroxysms/attacks/seizures, dizziness/vertigo, non-paroxysmal alterations of consciousness, movement disorders except paralysis, other sensory symptoms such as tingling sensations, problems of cognitive or communicative function, others). Sensory and motor deficits were put in a single category (and separated from other sensory symptoms or movement disorders), because from an emergency care perspective, they are viewed as potential stroke mimics. In the common case of multiple documented symptoms, the part of the clinical picture that led to the emergency presentation or that ultimately determined the first-line medical management decisions served as the basis for categorization. For example, if a patient had mild dizziness and tingling sensations followed directly by a prolonged convulsive seizure, the case was coded as “paroxysm/attack/seizure” and not as “dizziness/vertigo.” In addition, the acuity and course of presenting symptoms was recorded. The diagnosis at the end of emergency department care was extracted according to case records and its relation to FND was categorized as follows: FND; primarily probable/presumptive FND; functional symptoms as part of another non-functional neurological disorder (i.e. functional overlay, e.g. functional tremor in a patient with Parkinson’s disease; but not an isolated dissociative seizure in a patient with a history of epilepsy), or as part of a primary psychiatric disorder (e.g. tingling sensation during a panic attack; but not functional/dissociative seizures in a patient with anxiety disorder); unexplained clinical symptom without evidence of neurological disease (with or without FND mentioned as a possible differential diagnosis); non-functional symptoms/condition. The same categorization was also applied to the final diagnosis of patients who were admitted to inpatient neurological treatment. In these cases, we further noted whether the FND(-related) diagnosis was coded as primary diagnosis. In patients brought to the emergency department by an emergency physician, the preliminary diagnosis, treatment, and Glasgow Coma Scale scores were extracted.

### Statistical analysis

Demographic and clinical variables were summarized using frequencies, percentages, and medians with ranges. Changes in prehospital GCS between benzodiazepine-treated and untreated patients with functional/dissociative seizures were compared with a Mann–Whitney *U* test (*p* < 0.05).

### Ethics

This study was approved by the Ethics Committee of the Medical Faculty of the University Duisburg-Essen (Number 23-11,617-BO).

## Results

### Epidemiological characteristics

In 2023, 5504 neurological cases were treated in the emergency department. The keyword search of the entire emergency department documentation and further review identified 187 individual neurological emergency presentations that satisfied all inclusion criteria. The complementary inpatient discharge diagnosis-based search identified 18 additional cases. Of those 205 cases, 6 were ultimately diagnosed with a non-functional condition at discharge (1 discharged from ED, 5 from neurology wards). Thus, 199 patients, 3.6% of the total patient volume, had leading functional symptoms. Of those, 141 (71%) were female; age ranged between 18 and 83 (median: 33 years).

### Clinical characteristics

Most patients (90%) had acute-onset symptoms, while 8% presented with subacute problems (onset 2–30 days ago), and only 2% had chronically persistent symptoms. In total, 108 cases (54%) had a functional/dissociative seizure; 42 patients (21%) had a motor or sensory deficit; 18 (9%) had dizziness; 15 (8%) another sensory symptom; 12 (6%) had a movement disorder other than paralysis or weakness; in 4 patients (2%), the primary complaint was related to communication or cognitive problems.

Of all cases labelled as “seizures” at initial triage assessment (645 cases, 12% of total neurological cases), 14% (88/645) were ultimately diagnosed as functional (note that not all functional/dissociative seizures were initially triaged as “seizures”). For other symptom categories, such analyses were not feasible due to the lack of specificity of keywords used in the triage system (e.g. dizziness can be tagged as “unwell adult”, “fall”, “unspecified neurological problem” or “stroke”).

Overall, 154 (77%) received a confirmed or presumptive FND diagnosis; 12 (6%) were seen as functional neurological symptoms in the context of another neurological or psychiatric disorder (“functional overlay”); in 33 cases (17%), FND was considered a possible but not necessarily likeliest diagnosis or symptoms were simply “unexplained.”

### Management

**Fig. 1:**
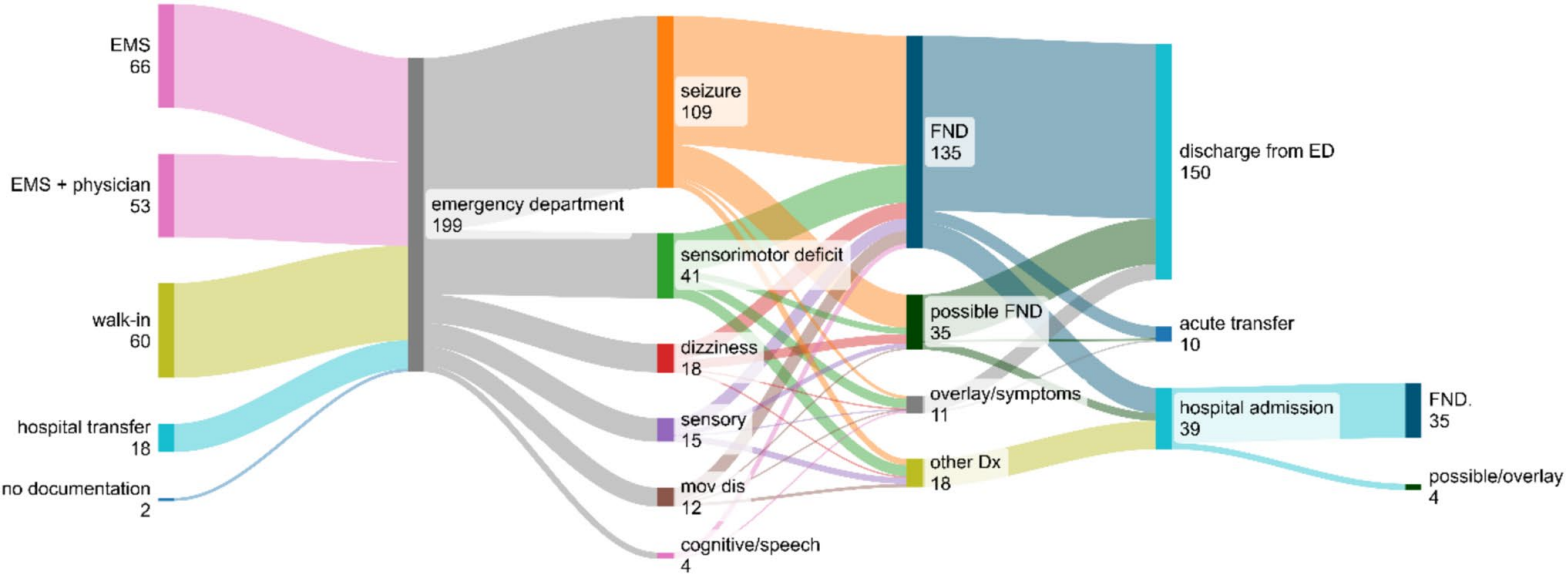
Sankey diagram shows presentation mode to the emergency department (ED), leading symptom as well as diagnostic classification before and after admission. *EMS* emergency medical services, ‘*seizure*’ denotes all functional paroxysms including prolonged loss of consciousness, ‘*sensory*’ refers to all functional symptoms except regional sensory deficit, ‘*mov dis*’ movement disorders except motor deficit (weakness/paralysis), *FND* confirmed or presumptive functional neurological disorder, ‘*possible FND*’ includes medically unexplained neurological symptoms, ‘*overlay/symptoms*’ refers to patients with functional neurological symptoms as part of an underlying non-functional neurological disease or a primary psychiatric disorder (see main text for details)

Most patients (60%) were brought by ambulance, about half of those accompanied by an emergency physician. Thirty percent were walk-ins and the rest were transferred from other departments. After acute treatment at the emergency department, 150 (75%) were discharged; 39 (19%) patients were admitted as inpatients; the remaining 10 patients were acutely transferred to another hospital or department (6 of those to psychiatry). Nine patients (4.5%) left the emergency department against medical advice, compared to 1.5% (83/5504) of all neurological ED cases in 2023. Of the 39 patients admitted as inpatients, 26 (67%) were admitted to the general neurology ward, while 13 (33%) were admitted to a monitoring unit (intensive care unit, stroke unit or epilepsy monitoring unit). Figure 1 shows an overview of treatment pathways, chief complaints, and diagnostic classification.

There were 37 cases with acute functional/dissociative seizures treated by emergency physicians on-site with available GCS scores. Of those, 7 patients had an initial GCS of 3, three of whom were intubated by the emergency physicians before arrival at the hospital. Of the 34 non-intubated patients, 13 received benzodiazepines during prehospital management. There was no significant difference in the change of GCS between first contact and handover between patients who received benzodiazepines and those who did not (Mann–Whitney *U* = 126, *p* = 0.72). Overall, the on-site intubation rate for patients with acute functional/dissociative seizures treated by emergency physicians was 7% (3/46).

### Diagnostic coding

Among the 111 patients that were discharged from the ED with FND or suspected/probable FND, only 43 (39%) had a F44 or F45 diagnostic coded in their discharge documentation. Among the 35 patients admitted as inpatients and discharged with FND or suspected/probable FND as a primary cause of their presenting complaints, 29 (83%) were coded as F44 or F45.

## Discussion

The main finding of our study is that at 3.6% functional symptoms represent a non-negligible component of all neurological emergency presentations, far exceeding the 0.4% reported for the U.S. in a database analysis that identified cases by ICD-10 codes alone. Furthermore, considering the high rates of misdiagnosis of FND across phenotypes [22], our findings should be taken as a minimal estimate of the true prevalence. A study from New Zealand determined that FND accounts for 9% of neurological emergency admissions, but did not consider ED visits that did not result in admission. Although 3.6% is not a large number, in practical terms, it means that FND will be a common if not daily occurrence in large emergency departments. This is accentuated by the fact that a quarter of these patients will involve prehospital treatment by emergency physicians with potentially life-threatening differential diagnoses such as stroke or status epilepticus, necessitating rapid and decisive workup in the emergency room. Thus, the recognition, assessment, and management particularly of acute-onset FND (accounting for 90% of presentations in this study) should be part of emergency medicine algorithms and curricula.

Discharge against medical advice was common among FND patients (4.5%, compared to background rate of 1.5%), suggesting a higher rate of dissatisfaction or disagreement with medical assessment. This is particularly salient, given that communicating the diagnosis in a way that is perceived as satisfactory by patients has measurable impacts on future health care utilization and costs [24]. Previous studies on discharge against medical advice from ED in neurological patients have not specifically investigated functional disorders. In a large chart review study from Germany, “non-neurological disorder” was the third most common diagnosis (after seizures and headaches) among patients who left the ED against medical advice or without notifying staff [25]. Discharge against medical advice is generally more common in seizure disorders such as epilepsy, likely because patients have experience with their paroxysmal symptoms and the postictal period, so feel less inclined to stay in hospital [26]. Thus, the higher rates in our FND cohort might also be attributable to an overrepresentation of paroxysmal disorders.

Functional/dissociative seizures are a major subtype of FND presenting through the emergency department. Previous studies have found that this represents a major challenge for hyperacute management with high rates of misdiagnosis and non-indicated treatment [19]. Although recognizing and diagnosing functional/dissociative seizures is often possible on clinical grounds, this type of semiological analysis is insufficiently disseminated among emergency physicians, leading to diagnostic uncertainty and unnecessary benzodiazepine treatment [18]. In a crude analysis of prehospital benzodiazepine treatment, we found no difference in GCS change compared to untreated patients. This is in line with recent guidelines to not administer benzodiazepines for functional/dissociative seizures [27]. The overall on-site intubation rate of 7% is comparable to other studies [18, 19, 28].

Inconsistent diagnostic classification and miscoding of FND is well established and contributes to an under-recognition of FND in retrospective studies [10, 20, 23, 29, 30]. An analysis based purely on documented ICD-10 codes would have picked up less than 40% of the total case count, in line with a similar study from Germany on inpatients [10], demonstrating the importance of prospective or hybrid case ascertainment strategies in epidemiological studies. This is added to known factors of under-recognition of FND. Viewed in this context, the already substantial and steeply rising annual costs of FND-related emergency care reported in the U.S. at an estimated incidence of 0.5% (> $2 billion) may significantly underestimate the true economic burden, which is likely several times higher when accounting for undiagnosed or miscoded cases [4, 5]. The high cost of FND stands at odds with the relatively minuscule research funding it receives [5].

This study has several limitations. First, it was conducted retrospectively at a single tertiary care center, limiting generalizability and relying on the completeness and accuracy of routine clinical documentation. Our case ascertainment focused on adult neurological ED presentations, potentially underestimating the true burden of FND by excluding pediatric, surgical, or other non-neurological pathways. Despite using a hybrid search strategy, some cases may have been missed due to inconsistent terminology or undocumented diagnostic impressions. Diagnostic certainty was constrained by the absence of long-term follow-up, although we applied established criteria and cross-checked documentation to mitigate this. Without a comparator group of non-FND patients, relative differences in clinical pathways, resource use, or outcomes could not be systematically assessed. In some analyses, we included cases whose neurological symptoms were simply “unexplained” (without evidence of neurological disease), which is not equatable to FND and introduces a degree of imprecision. Lastly, there are ongoing debates around the diagnostic taxonomy of FND, such as whether there is such a thing as functional overlay (e.g. functional weakness in a stroke patient) different from comorbidity (functional/dissociative seizures in a patient with epilepsy), or when a functional neurological symptom is considered an independent disorder or part of another psychiatric condition (e.g. dissociative symptoms in a patient with complex posttraumatic stress disorder). In this regard, our choice of categories is arbitrary and might bias our findings.

In conclusion, our study demonstrates that FND is a relatively common reason for seeking emergency medical help, confirming the previously noted necessity for improving clinical training, developing specific management pathways, and funding further research [31]. Improving prehospital and ED management of common subtypes such as functional/dissociative seizures is of key importance. Greater diagnostic clarity and confidence as well as unambiguous coding practices will be necessary to uncover the true impact of FND in a range of settings and adjust health care services and attitudes accordingly.

## Data Availability

The retrospective clinical data underlying this study are not publicly available due to privacy regulations, but anonymized data can be made available from the corresponding author upon reasonable request.
